# LPS mediates cuproptosis and inflammation in THP-1 macrophages through HKDC1: LPS mediates cuproptosis and inflammation in THP-1 macrophages

**DOI:** 10.3724/abbs.2025089

**Published:** 2025-07-22

**Authors:** Langlin Ou, Zitong Meng, Jian Mei, Hao Yuan, Xiangrui Zhu, Xiaoying Wang, Ao Shen, Zhaosi Wang, Lixin Zhang, Song Wang, Yingli Chen, Xiangming Pang, Yuxiang Liu, Yadong Xu, Cui Ma

**Affiliations:** 1 College of Medical Laboratory Science and Technology Harbin Medical University Daqing 163319 China; 2 Institute of Cardiovascular Diseases Xiamen Cardiovascular Hospital School of Medicine Xiamen University Fujian Branch of National Clinical Research Center for Cardiovascular Diseases Xiamen 361102 China; 3 College of Pharmacy Harbin Medical University Harbin 150081 China; 4 College of Pharmacy Harbin Medical University Daqing 163319 China; 5 Basic Medical College Harbin Medical University Daqing 163319 China; 6 Department of Pulmonary and Critical Care Medicine Daqing People’s Hospital Daqing 163316 China

**Keywords:** LPS, inflammation, glycolysis, HKDC1, cuproptosis

## Abstract

Cuproptosis is a recently identified form of copper-driven cell death characterized by the aggregation of acylated proteins and proteotoxic stress in the mitochondrial tricarboxylic acid cycle, which plays a role in inflammation. Recent studies suggest that hexokinase structural domain protein 1 (HKDC1), a fifth hexokinase, is involved in regulating mitochondrial function. However, the role of HKDC1 in cuproptosis and LPS-induced macrophage inflammation remains unclear. Here, we assess macrophage plasticity using CCK8 viability assays and phagocytosis activity experiments in an *in vitro* inflammatory model of THP-1 cells. We measure the levels of inflammatory factors and cuproptosis-related proteins using western blot analysis and RT-qPCR. Additionally, we examine the expression and localization of the HKDC1 protein using ChIP-qPCR and immunofluorescence staining. We find that LPS promotes the expressions of inflammatory factors and decreases cuproptosis levels in THP-1-derived macrophages while also activating glycolysis and inducing the expression of HKDC1 via the Toll-like receptor 4 (TLR4) receptor. We further demonstrate that *HKDC1* knockdown inhibits glycolysis and induces cuproptosis. Mechanistically, we provide the first evidence that LPS promotes the binding of Yin Yang 1 (YY1) to the *HKDC1* promoter, thereby regulating *HKDC1* transcription. HKDC1 interacts with heat shock cognate B (HSCB) and ferredoxin 1 (FDX1), leading to increased intracellular copper levels and subsequent cuproptosis. *HKDC1* knockdown *in vivo* alleviates acute sepsis by activating copper-dependent cell death pathways. Collectively, our findings suggest that LPS mitigates cuproptosis and promotes inflammation via HKDC1, suggesting a new cuproptosis-dependent anti-inflammatory strategy.

## Introduction

Inflammation is a vital component of a series of reactions, such as cell dysfunction and immune abnormalities triggered by various factors, including ischemia and infection [1]. The role of inflammation in promoting the occurrence and progression of diseases, such as pulmonary vascular inflammation caused by hypoxia, chronic obstructive pulmonary disease (COPD), and acute respiratory distress syndrome (ARDS), has been demonstrated [ 2– 4]. Long-term inflammation of the liver and rectum increases the risk of hepatocellular carcinoma and colorectal cancer, respectively [5]. Additionally, inflammation is a key pathogenic factor of cardiovascular and cerebrovascular diseases [6]. Macrophages play a central role in the development of chronic inflammation and excessive scarring by driving and resolving inflammation and restoring tissue homeostasis [7]. The THP-1 cell line, which was treated with phorbol myristate acetate (PMA; 100 ng/mL) and stimulated with lipopolysaccharide (LPS), is widely used to polarize M1 macrophages and serves as a typical inflammatory model in research on aging, pulmonary hypertension, and cancer [ 8– 10]. In this study, THP-1-derived macrophages stimulated with LPS were used to further explore the diversity of macrophages and their contributions to inflammatory responses.

Glycolysis, the fundamental process of glucose metabolism, is catalyzed by hexokinase and pyruvate kinase, which decompose glucose into pyruvate and produce ATP for the body [11]. Hexokinase domain containing 1 (HKDC1) is a novel hexokinase that is widely expressed in various tissues in humans and mice [ 12, 13]. Previous studies have demonstrated that aberrantly expressed HKDC1 plays an essential role in the progression of malignant tumors, such as lymphoma and liver, breast, and colorectal cancers [ 13– 16]. Moreover, Cui *et al*. [17] reported that HKDC1 is critical for maintaining mitochondrial homeostasis by regulating the PINK1/Parkin-dependent pathway and is important for maintaining mitochondria-lysosome contact. However, the effects of HKDC1 on inflammation in macrophages and the underlying mechanisms remain unclear.

Cuproptosis is a recently discovered type of copper-dependent cell death [ 17, 18]. Copper dyshomeostasis leads to Cu ^2+^ overload. Ferredoxin 1 (FDX1), a reducing protein, converts Cu ^2+^ to the more toxic Cu ^+^ [18]. Cu ^+^ subsequently causes abnormal lipoylation and aggregation of lipoyl synthase (LIAS), a lipoylation-related enzyme and a key component of pyruvate dehydrogenase. This process also results in the loss of iron-sulfur (Fe-S) cluster proteins. Moreover, Cu ^+^ directly binds to lipid components in the tricarboxylic acid cycle, leading to an increase in the expression of the heat shock protein Hsp70 [ 17– 19]. Oxidative damage to the mitochondria and impairment of the tricarboxylic acid cycle subsequently result in acute toxic stress on proteins, ultimately leading to cell death [20]. Numerous studies have shown that various modes of cell death, including apoptosis, pyroptosis, and ferroptosis, can influence disease progression by modulating the intensity and duration of inflammatory responses [ 21– 23]. Although cuproptosis has been reported to be associated with rheumatoid arthritis, periodontitis inflammation, and neuroinflammatory development [ 24– 27], the potential regulatory mechanisms of cuproptosis in inflammation remain to be elucidated and require further exploration.

In the present study, we aimed to determine whether HKDC1 can regulate cuproptosis through glycolytic modulation under stress conditions. We found that LPS promoted the expressions of inflammatory factors and decreased the degree of cuproptosis in THP-1-derived macrophages. This occurs alongside the activation of glycolysis and the induction of HKDC1 expression. Knockdown of *HKDC1* inhibited the glycolytic pathway and reduced the binding of heat shock cognate B (HSCB) to ferredoxin 1 (FDX1), thereby promoting the expressions of cuproptosis-related proteins, including FDX1, lipoic acid synthase (LIAS), and Hsp70. Collectively, our findings demonstrated for the first time that LPS resists cuproptosis and promotes inflammation through the HKDC1-regulated glycolytic pathway.

## Materials and Methods

### Cell culture

THP-1 cells (No. CL-0233) were obtained from Procell Life Science & Technology (Wuhan, China). These cells were maintained in RPMI 1640 culture medium (BL303A; Biosharp, Wuhan, China) containing 15% FBS and 1% penicillin-streptomycin at 37°C with 5% CO _2_, and 100% relative humidity. PMA was dissolved in DMSO and diluted to a final concentration of 0.1 μg/mL. The cells were then treated with PMA (100 ng/mL) and plated into 6-well plates at 2 mL per well, followed by induction for 48 h. Some cells were pretreated with the TLR4 inhibitor Resatorvid (TAK-242, 200 nM; HY-11109; MCE, Shanghai, China) for 1 h before LPS (L8880; Solarbio, Beijing, China) induction.

### Cell transfection

Prior to transfection, the cells were seeded into 6-well or 12-well plates and maintained at a growth density of 70%–80% confluence. Small interfering RNAs (siRNAs) targeting *HKDC1* and *YY1* (Yin Yang 1), and a nontargeted control (NC) were designed and synthesized by GenePharma (Suzhou, China). The sequences are shown in Table 1. The siRNAs were subsequently transfected into THP-1 cells using Lipofectamine 2000 reagent (11668019; Thermo Fisher Scientific, Waltham, USA) following the manufacturer’s instructions.

**Table:** **Table 1** siRNA sequences used in this study

Gene	Sense (5′→3′)	Antisense (5′→3′)
NC	UUCUCCGAACGUGUCACGUTT	ACGUGACACGUUCGGAGAATT
si-HKDC1	CAGUGCGAAUGUACAACAATT	UUGUUGUACAUUCGCACUGTT
si-YY1	CCAAACAACUGGCAGAAUUTT	AAUUCUGCCAGUUGUUUGGTT

### Cell Counting Kit 8 assay

For the CCK-8 assay, THP-1 cells were treated with PMA (100 ng/mL) and plated into 96-well plates at a density of 3 × 10 ^4^ cells per well. The 2-deoxy-D-glucose (2-DG) concentration gradient was then designed on the basis of concentrations reported in previous studies [ 28– 30]. The cells were pre-treated with fresh medium containing 0, 0.5, 1, 5, 10, or 20 mM 2-DG or LPS for 24 h. Afterward, 10 μL of Cell Counting Kit 8 reagent (C0042; Beyotime, Shanghai, China) was added to each well and incubated for 2 h at 37°C. The absorbance was measured at 450 nm using a microplate reader (SpectraMax ABS plus; Molecular Devices, Shanghai, China). The 10 mM 2-DG that significantly inhibited cell viability was used in the subsequent experiments.

### RNA isolation and quantitative real-time polymerase chain reaction (RT-qPCR)

Total RNA was extracted from mouse tissues and THP-1 macrophages cultured under different conditions using TRIzol reagent (R0011; Beyotime). The RNA was then reverse transcribed into cDNA using the Golden 1st cDNA Synthesis kit (D0401; Hai Gene, Harbin, China). To quantify the RNA levels, qPCR was performed on a LightCycler 480 II system (Roche, Basel, Switzerland) using SYBR Green (QPK-201; TOYOBO, Osaka, Japan). The threshold cycle (Ct) values were determined, and the mRNA levels were quantified using the 2 ^–ΔΔCT^ method. The primers used are listed in Table 2.

**Table:** **Table 2** Sequences of primers used for RT-qPCR in this study

Gene	Forward primer (5′→3′)	Reverse primer (5′→3′)
Human- *actin*	CCTCCCTCTGGCACACACTTG	TGTGTTGGCGTACAGGTCTT
Mouse- *actin*	CTGTCCCTGTATGCCTCTG	ATGTCACGCACGATTTCC
*HKDC1*	TGTCTGTACCATCGTCTCCTTCC	GCCGTTCCACCTTCTTGTTCTC
*HKDC1*-promoter	AACCCATCAATACCCTGCTTCCC	CCTCCCTCTGGCACACACTTG
Human- *IL-1β*	AGGCTGCTCTGGGATTCTCTT	TGGTGGTCGGAGATTCGTAG
Mouse- *IL-1β*	GCCACCTTTTGACAGTGATGAG	AAGGTCCACGGGAAAGACAC
Human- *IL-6*	CCACCGGGAACGAAAGAGAA	GAGAAGGCAACTGGACCGAA
Mouse- *IL-6*	GTCCTTCCTACCCCAATTTCCA	TAACGCACTAGGTTTGCCGA
Human- *TNF-α*	GGCGTGGAGCTGAGAGATAA	AGTCGGTCACCCTTCTCCAG
Mouse- *TNF-α*	ATGGCCTCCCTCTCATCAGT	TTTGCTACGACGTGGGCTAC
Human- *MYD88*	CGGATGGTGGTGGTTGTCTCTG	GCTGGGGAACTCTTTCTTCATTGC

### Western blot analysis

Total protein was extracted from mouse tissues and PMA (100 ng/mL)-treated THP-1 cells using RIPA buffer (P0013B; Beyotime). The proteins were equally loaded and separated by SDS-PAGE (8%–12%). Proteins were then transferred to a nitrocellulose membrane (Millipore, Billerica, USA). After being blocked with 5% non-fat milk, the membranes were incubated overnight at 4°C with primary antibodies specific for the targets listed in Table 3. Following washing in TBST buffer containing Tween-20, the membranes were incubated with horseradish peroxidase-coupled secondary antibodies for 1 h at room temperature. The blots were then imaged using enhanced chemiluminescent reagents.

**Table:** **Table 3** List of primary antibodies

Antibody name	Working concentration	Catalogue #	Vendor or source
FDX1	1:500	M05441	Boster, Wuhan, China
Hsp70	1:500	BM4335	Boster
LIAS	1:500	11577-1-AP	Proteintech, Wuhan, China
HK2	1:4000	66974-1Ig	Proteintech
YY1	1:1000	22156-1-AP	Proteintech
PKM2	1:500	4053	Cell-Signalling, Danvers, USA
HKDC1	1:1000	A16573	ABclonal, Wuhan, China
NF-κB	1:500	A11204	ABclonal
HSCB	1:500	A15961	ABclonal
IL-1β	1:500	PTM-5116	PTM BIO, Chicago, USA

### Measurement of copper (Cu ^2+^) concentration

THP-1 cells were plated in 6-well plates overnight and treated according to the different experimental groups for 24 h. The cells were subsequently collected and resuspended in 120 μL of distilled water. The cells were disrupted by ultrasound (E-BC-K775-M; Elabscience, Wuhan, China) following the manufacturer’s instructions. The cell supernatant was subsequently collected for Cu ^2+^ detection. The Cu ^2+^ concentration in mouse liver tissue was detected using a copper (Cu) colorimetric assay kit (E-BC-K300-M; Elabscience) according to the manufacturer’s instructions. Briefly, 15 μL of the liver tissue lysate sample was mixed with 230 μL of the chromogenic agent working solution and incubated at 37°C for 5 min. Then the absorbance was detected at a wavelength of 580 nm using a microplate reader (SpectraMax ABS plus).

### Phagocytosis assay

A Vybrant phagocytosis assay kit (#V6694; Thermo Fisher Scientific) was used to assess the phagocytic ability of the THP-1 cells. Briefly, THP-1 cells were seeded in 96-well plates and treated according to the different experimental groups for 24 h prior to the phagocytosis assay. After the original medium was removed, 100 μL of fluorescein-labelled *Escherichia coli* K-12 bacteria was added, and the cells were incubated at 37°C in the dark for 2 h. The *E*. *coli* particles were subsequently aspirated, and the cells were incubated with trypan blue at room temperature for 1 min. Finally, the uptake level of the fluorescent particles was evaluated at an excitation wavelength of 480 nm and an emission wavelength of 520 nm. The phagocytic activity of the THP-1 cells was calculated using the following formula: phagocytosis effect% = (net experimental value/net phagocytic value) × 100%.

### Measurement of the intracellular pyruvate concentration

THP-1 cells were seeded in 6-well plates and incubated overnight. The cells were then treated according to the different experimental groups for 24 h. Approximately 1 × 10 ^4^ cells were subsequently collected in a centrifuge tube, and the supernatant was discarded after centrifugation. The cells were lysed via ultrasonication, and the pyruvate concentration in the cell supernatant was measured using a pyruvate assay kit (BC2205; Solarbio) following the manufacturer’s instructions.

### Measurement of intracellular pyruvate dehydrogenase activity

THP-1 cells were seeded in 6-well plates overnight and then treated according to the different experimental groups for 24 h. First, approximately 5 × 10 ^6^ cells were collected in a centrifuge tube, and the supernatant was discarded after centrifugation. The cells were lysed by ultrasonication, and the pyruvate dehydrogenase activity in the cell supernatant was measured using a pyruvate dehydrogenase detection kit (BC0380; Solarbio) according to the manufacturer’s instructions. Finally, the absorbance was detected at a wavelength of 605 nm using a microplate reader (SpectraMax ABS plus).

### Immunofluorescence staining

THP-1 cells from different groups were fixed using 4% paraformaldehyde for 15 min and permeabilized with 0.3% Triton X-100 for 10 min. The cells were washed with pre-cooled PBS and incubated with an immunostaining blocking solution for 30 min at room temperature. The cells were then incubated with an anti-HKDC1 antibody (1:100; A16573; ABclonal) at 4°C overnight. The next day, the cells were washed and incubated with FITC-coupled secondary antibodies for 2 h at 37°C in the dark. For tissue fluorescence experiments, after deparaffinization, permeabilization, and serum blocking of 5-μm-thick tissue sections, the tissue sections were incubated with an anti-CD68 antibody (1:50; GB113109-100; Servicebio) at 4°C overnight. The following day, the sections were washed and incubated with fluorescent-labeled secondary antibodies for 2 h at 37°C in the dark. Finally, the cells or tissue sections were stained with 4ʹ,6-diamidino-2-phenylindole (DAPI) for 10 min. The results of the immunofluorescence staining were examined with a live cell workstation (AF6000; Leica, Wetzlar, Germany).

### Chromatin immunoprecipitation quantitative PCR

A chromatin immunoprecipitation (ChIP) assay was conducted using a ChIP assay kit (P2078; Beyotime). Briefly, THP-1 cells were seeded into 10-cm culture dishes and incubated overnight. They were then cross-linked with a final concentration of 1% formaldehyde solution at 37°C for 10 min. The cells were subsequently incubated with a 10× glycine solution at room temperature for 5 min to neutralize the formaldehyde. Chromatin fragments, ranging from 100 bp to 1000 bp, were treated with SDS and PMSF and then sonicated. Antibodies against YY1 and normal rabbit IgG were used for immunoprecipitation of the fragmented chromatin. The cross-links between histone and DNA were subsequently removed by incubation at 65°C for 2 h, and the chromatin was purified using a DNA purification kit (D0033; Beyotime). Finally, RT-qPCR was performed to detect the enrichment of YY1 in the *HKDC1* promoter fragment. The primers used are shown in Table 2.

### Protein co-immunoprecipitation (Co-IP)

During the protein Co-IP experiment, THP-1 cells were first lysed, and the cell contents were collected. The cell supernatant was then retained after centrifugation at 13,000 *g* for 30 min at 4°C. Next, 5 μg of the target antibody or IgG was added to the supernatant, and the mixture was incubated on a shaker at 4°C for 6 h. Subsequently, protein A+G agarose beads (P2055; Beyotime) were added, and the mixture was incubated on a shaker at 4°C overnight. The next day, the antibody-protein complex was washed, and 2× protein loading buffer was added to the beads, which were subsequently mixed. Finally, western blot analysis was performed.

### Animal treatments and H&E staining

All animal experiment procedures used in this study were in accordance with the 1964 Helsinki Declaration and its subsequent revisions and were approved by the Ethics Committee of Harbin Medical University (HMUDQ20241214001). C57BL/6 mice, aged 6–8 weeks and weighing 20–25 g, were purchased from the Laboratory Animal Center of the Second Affiliated Hospital of Harbin Medical University (Harbin, China). The RNAs targeting *HKDC1* and the serotype 9 adenovirus-associated virus (AAV9) were designed and synthesized by Genechem (Shanghai, China). The mice were randomly divided into 3 groups: the PBS + AAV9-NC group, the LPS + AAV9-NC group, and the LPS + AAV9-shHKDC1 group. C57BL/6 mice were injected with 200 μL of AAV9-shHKDC1 [30] (target sequence: 5′-CAATGAAATCACCCGTGGGAA-3′) or AAV9-shNC (5′-CGCTGAGTACTTCGAAATGTC-3′) at a dose of 3.0 × 10 ^11^ genomic equivalents through the caudal vein. Twenty days later, an intraperitoneal injection of LPS (5 mg/kg; L8880; Solarbio) was used to establish an acute sepsis model, as described in previous studies [ 31 , 32]. The animals were euthanized 24 h after LPS stimulation, and serum, lung, liver, and kidney tissues were collected for further analysis. For H&E staining, the left lung and liver tissues from the mice were fixed in 4% paraformaldehyde, embedded in paraffin, and sectioned at a thickness of approximately 5 μm. These sections were then stained with hematoxylin and eosin and examined under a microscope.

### Retrieval of transcription factor (TF) annotation information database

The annotations of transcription factors (TFs) were retrieved from the PROMO ( https://alggen.lsi.upc.es/cgibin/promo_v3/promo/promoinit.cgi?dirDB=TF_8.3), Ominer ( http://signalingpathways.org/ominer/query.jsf), and AnimalTFDB ( https://ngdc.cncb.ac.cn/databasecommons/database/id/8) databases. The results from these three databases were cross-analyzed using the Venny 2.1 online tool to generate a Venn diagram. Known TF binding motifs were obtained from the JASPAR database ( https://jaspar.genereg.net/). Specifically, the Homo sapiens species was selected on the JASPAR homepage. After querying relevant transcription factor information, the SCAN function was used to input the *HKDC1* gene promoter sequence, with the relative profile score threshold set to 90%, ultimately yielding high-confidence transcription factor binding sites and their corresponding binding scores.

### Statistical analysis

All the experiments were repeated at least three times, and the quantitative data are presented as the mean ± standard deviation (SD). The experimental data were statistically processed and graphed using GraphPad Prism 8 software. Before the statistical test, the normal distribution and F test (equal variance test) were performed on each group of data. Unpaired Student’s *t* tests were used to compare the parameters between two groups with equal variance, and the Welch correction test was used to compare the parameters between the two groups with unequal variance. One-way ANOVA with Tukey’s post hoc test was used to compare the parameters of more than two groups with equal variance, and BrownForsythe and Welch ANOVA with Tamhane’s T2 post hoc test were used to compare the parameters of more than two groups with unequal variance. Nonparametric analysis was performed on data that did not conform to a normal distribution, including the Mann-Whitney U test for two groups of parameters or the Kruskal-Wallis test followed by the Dunn post hoc test for more than two groups of parameters. *P* < 0.05 was considered statistically significant.

## Results

### LPS mediates the inflammatory response by regulating cuproptosis

**Figure 1:**
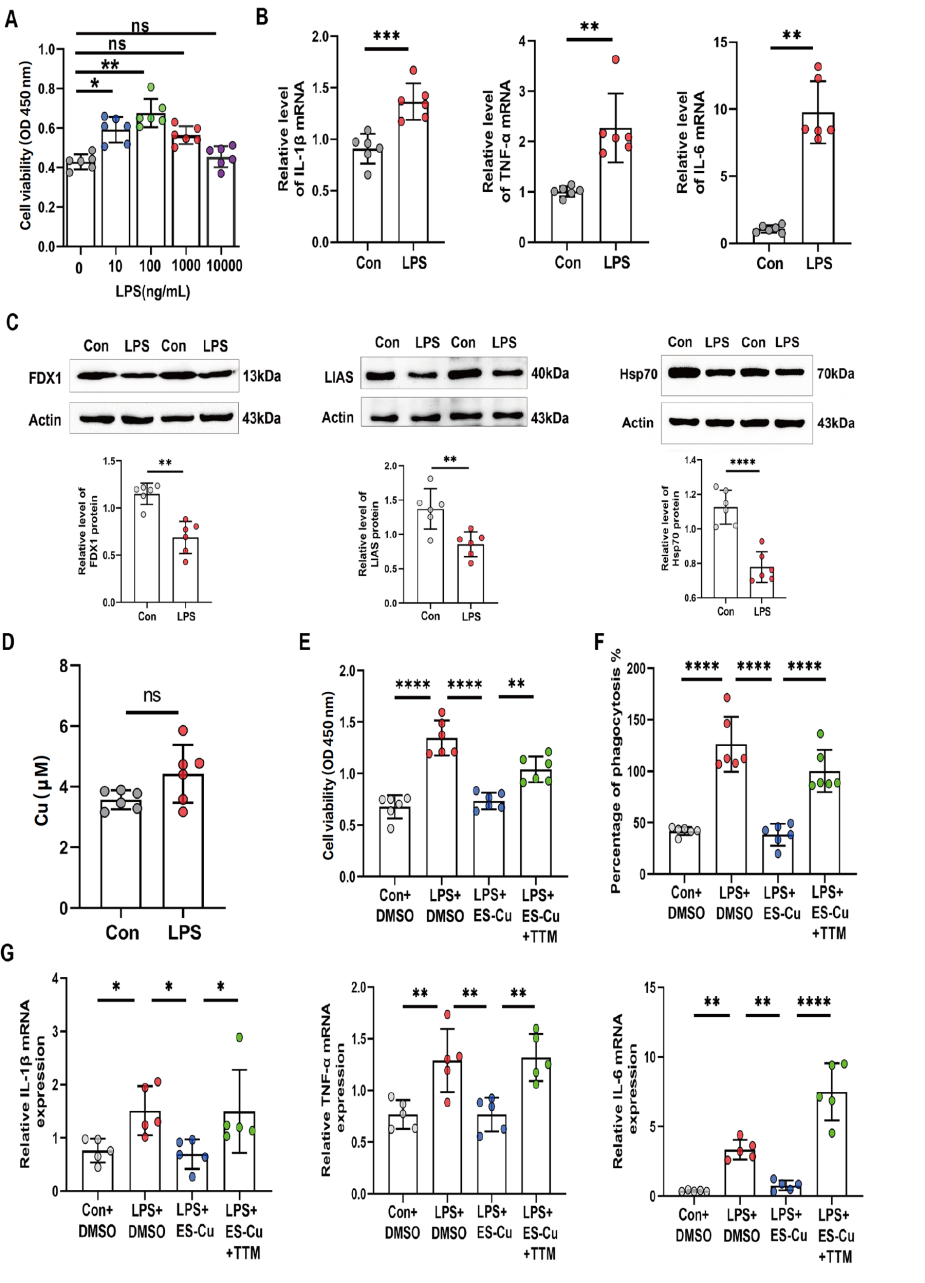
Figure 1 LPS prevents cuproptosis and promotes the expression of inflammatory factors (A) CCK-8 assay was used to detect cell viability after stimulation with different concentrations of LPS (n = 6). (B) RT-qPCR was used to detect the mRNA levels of IL-1β, TNF-α and IL-6 after LPS treatment (n = 6). (C) Western blot analysis was used to detect the expressions of FDX1, LIAS, and Hsp70 (n = 6). (D) Intracellular copper ion levels were detected (n = 6). (E) The viability of THP-1 cells was assessed after different experimental treatments ( n = 6). (F) Phagocytosis assay was used to detect the phagocytic activity of THP-1 cells under different experimental conditions (n = 6). (G) RT-qPCR was used to detect the mRNA levels of IL-1β, TNF-α, and IL-6 (n = 5). Data are presented as the mean ± SD. One-way ANOVA with Tukey’s post hoc test was used for multiple comparisons, and independent samples t tests were employed for pairwise comparisons. Con, control. ES-Cu, Elesclomol, and CuCl 2 were mixed at ratios of 1:1 and 100 nM. TTM, Tetrathiomolybdate, 10 μM. *P < 0.05, **P < 0.01, *** P < 0.001, ****P < 0.0001.

CCK-8 assay was conducted to detect the effects of various concentrations of LPS (0, 10, 100, 1000, and 10,000 ng/mL) on THP-1 cell viability after 24 h of treatment. The results revealed that cell viability was highest at an LPS concentration of 100 ng/mL ( Figure 1A). Additionally, RT-qPCR analysis revealed that 100 ng/mL LPS upregulated the expressions of inflammatory factors ( Figure 1B). Consequently, we chose 100 ng/mL LPS as the optimal condition for inducing inflammation in subsequent experiments. We then attempted to elucidate the correlations between LPS and cuproptosis. Western blot analysis revealed that LPS treatment inhibited the expressions of key cuproptosis proteins, including FDX1, LIAS, and Hsp70, in THP-1 macrophages ( Figure 1C). Additionally, intracellular Cu ^2+^ concentration assay revealed that while LPS induced a slight increase in the Cu ^2+^ level, this increase was not statistically significant ( Figure 1D). Next, we investigated the effect of cuproptosis on THP-1 cell viability. CCK-8 experiments revealed that the LPS-induced increase in cell viability was inhibited by the cuproptosis activator ES-Cu. In contrast, cell viability was rescued by inhibiting copper-binding proteins with tetrathiomolybdate (TTM) ( Figure 1E). We further assessed phagocytic function. Compared with those in the LPS group, the phagocytic function of the cells treated with ES-Cu (represented as a percentage of phagocytosis) was significantly reduced. In contrast, the phagocytic function of TTM-treated cells was significantly restored compared with that of the LPS + ES-Cu group ( Figure 1F). Finally, RT-qPCR experiments revealed that the mRNA levels of *IL-1β*, *TNF-α*, and *IL-6* were significantly reduced in the LPS combined with the cuproptosis activator ES-Cu treatment group. Conversely, the mRNA expressions of these inflammatory factors were restored in the LPS + ES-Cu + TTM group ( Figure 1G). These results suggest that LPS promotes cell viability and accelerates the release of inflammatory factors by inhibiting cuproptosis in THP-1 macrophages.

### LPS/TLR4 regulates glycolysis and its key gene *HKDC1*

**Figure 2:**
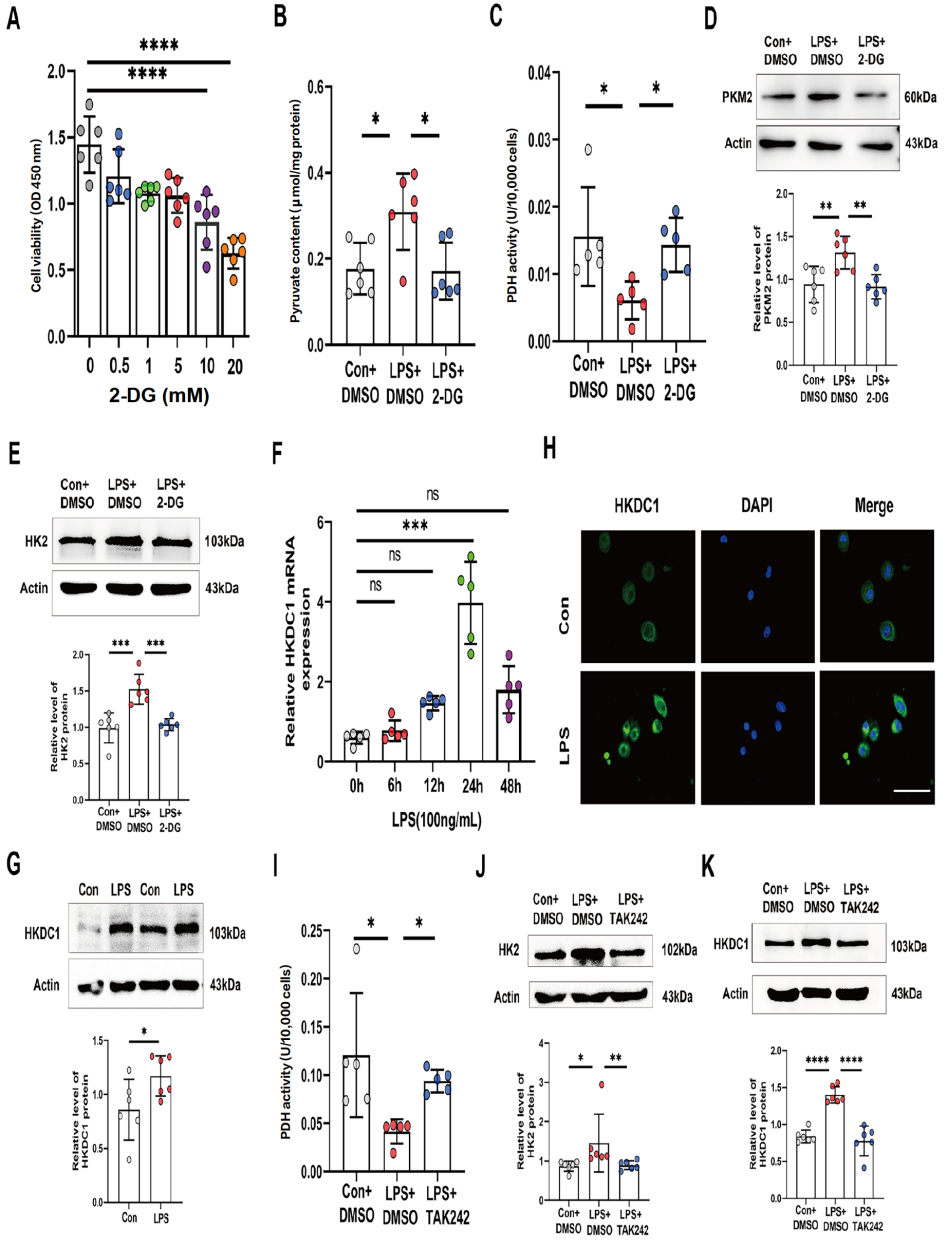
Figure 2 LPS promotes glycolysis and the expression of its key gene, *HKDC1*, through TLR4 (A) CCK-8 assay was used to detect the viability of cells stimulated with different concentrations of 2-DG (n = 6). (B,C) Detection of PA content (B) (n = 6) and PDH activity (C) (n = 5) under LPS and 2-DG stimulation. (D,E) Western blot analysis was used to detect the expressions of PKM2 and HK2 (n = 6). (F) RT-qPCR was used to detect the mRNA level of HKDC1 (n = 5). (G) Western blot analysis was used to detect the expression of HKDC1 (n = 6). (H) Immunofluorescence staining was used to detect the intracellular localization and expression of HKDC1 (n = 3). Scale bar, 50 μm. (I) Detection of PDH activity under LPS and TAK-242 stimulation ( n = 5). (J,K) Western blot analysis was used to detect the expressions of HK2 and HKDC1 under LPS and TAK-242 stimulation (n = 6). Data are presented as the mean ± SD. One-way ANOVA with Tukey’s post hoc test was used for multiple comparisons, and independent samples t tests were employed for pairwise comparisons. 2-DG, 2-Deoxy-D-glucose. The cells were pre-treated with 200 nM TAK-242 for 1 h. Con, control. ns not significant, *P < 0.05, **P < 0.01, *** P < 0.001, ****P < 0.0001.

Previous studies have demonstrated that the activation of glycolysis can prevent the onset of cellular cuproptosis [ 19, 33]. To investigate whether LPS regulates cuproptosis through glycolysis in macrophages, the cells were initially treated with the glycolysis inhibitor 2-DG. The CCK8 assay results indicated that cell viability was significantly reduced at a concentration of 10 mM 2-DG, which was determined to be the optimal concentration ( Figure 2A). Compared with the control, LPS increased the intracellular pyruvate content (PA) and decreased pyruvate dehydrogenase (PDH) activity in THP-1 cells. Treatment with 2-DG reversed these effects ( Figure 2B,C). Western blot analysis revealed that 2-DG treatment inhibited the LPS-induced expressions of pyruvate kinase M2 (PKM2) and hexokinase 2 (HK2) proteins ( Figure 2D,E). Next, we verified that LPS increased the expression of the glycolysis regulatory gene *HKDC1* using RT-qPCR. We found that *HKDC*1 expression peaked after 24 h of LPS stimulation ( Figure 2F). Additionally, western blot analysis and cellular immunofluorescence staining experiments revealed that LPS promoted HKDC1 protein expression primarily in the cytoplasm ( Figure 2G,H). Previous studies have demonstrated that LPS is recognized by Toll-like receptor 4 (TLR4) and triggers the secretion of inflammatory cytokines in macrophages and other immune cells via TLR signaling pathways [ 34– 36]. To demonstrate how LPS induces the activation of glycolysis, we treated THP-1 cells with the TAK-242. The results revealed that LPS significantly increased HK2 expression and decreased PDH activity, effects that were reversed by TAK-242 ( Figure 2I,J). Additionally, the western blot analysis results demonstrated that TAK-242 treatment suppressed the protein expression of HKDC1 induced by LPS ( Figure 2K). These findings indicate that LPS may promote cellular glycolysis and the expression of the key gene *HKDC1* by binding to the TLR4 receptor, thereby leading to intracellular stress.

### LPS mediates cuproptosis via HKDC1

**Figure 3:**
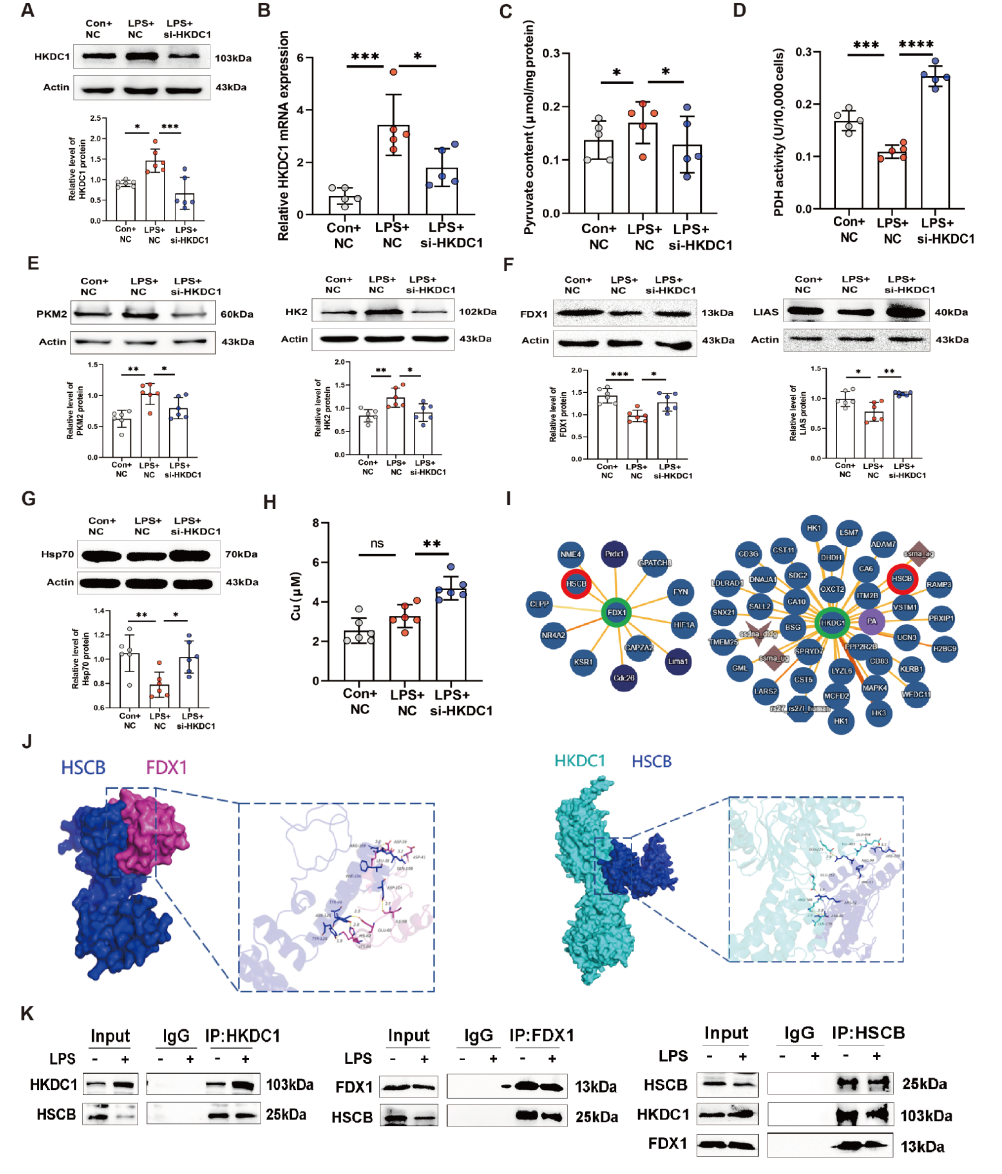
Figure 3 si-HKDC1 reverses glycolysis-dependent cell cuproptosis inhibited by LPS (A,B) Western blot analysis (n = 6) and RT-qPCR (n = 5) were used to detect the efficiency of HKDC1 interference. (C,D) PA content and PDH activity were measured after si-HKDC1 treatment (n = 5). (E) Western blot analysis was used to detect the expressions of PKM2 and HK2 after si-HKDC1 treatment (n = 6). (F,G) Western blot analysis was used to detect the protein expressions of FDX1, LIAS, and Hsp70 after si-HKDC1 treatment (n = 6). (H) Intracellular copper content was measured after si-HKDC1 treatment (n = 6). (I) The IntAct Molecular Interaction Database (https://www.ebi.ac.uk/intact/home) was utilized to screen for proteins that interact with FDX1 and HKDC1. (J) The HDOCK server (http://hdock.phys.hust.edu.cn/) was used to predict the binding interactions between FDX1, HKDC1, and HSCB. (K) Co-IP experiments confirmed the binding interactions among FDX1, HKDC1, and HSCB. Data are presented as the mean ± SD. One-way ANOVA with Tukey’s post hoc test was used for multiple comparisons, and independent samples t tests were employed for pairwise comparisons. Con, control. NC, negative control. si-HKDC1, siRNA-mediated HKDC1 knockdown. Input, whole-cell lysate. IgG, nonspecific binding control. IP, immunoprecipitated protein complex. ns, not significant. *P < 0.05, **P < 0.01, *** P < 0.001, ****P < 0.0001.

To determine the role of HKDC1 in LPS-regulated cuproptosis, the RNA interference fragment of HKDC1 was first transfected into THP-1 cells. The results revealed that, compared with NC, si-HKDC1 significantly reduced HKDC1 protein and mRNA expressions ( Figure 3A,B). We found that LPS increased the intracellular PA content, which was decreased by si-HKDC1 ( Figure 3C). Moreover, the trend in intracellular PDH activity was opposite to that in PA activity ( Figure 3D). The western blot analysis results revealed that si-HKDC1 decreased the expressions of PKM2 and HK2 induced by LPS in THP-1 cells ( Figure 3E). These results indicate that LPS regulates glycolysis through HKDC1. To further explore the association between HKDC1 and cuproptosis, we found that si-HKDC1 alleviated the LPS-induced decrease in the expressions of cuproptosis-related proteins, including FDX1, LIAS, and Hsp70, as determined by western blot analysis ( Figure 3F,G). In addition, compared with the LPS + NC group, the LPS + si-HKDC1 group presented a significantly greater concentration of Cu ^2+^ in the THP-1 cells ( Figure 3H). To investigate whether HKDC1 regulates the expressions of copper poisoning-related proteins through direct or indirect mechanisms, we first conducted bioinformatics analysis to predict the binding of HKDC1 and FDX1. Although HKDC1 and FDX1 have weak binding abilities, HSCB (heat shock cognate B) has a strong affinity for both HKDC1 and FDX1 ( Figure 3I). HSCBs are chaperone proteins that facilitate the recruitment of Fe-S cluster complexes, thereby maintaining cellular energy metabolism homeostasis [ 37– 40]. Therefore, we speculate that HSCBs may play a regulatory role in FDX1-related cuproptosis. We subsequently performed molecular docking analysis on HKDC1, FDX1, and HSCB ( Figure 3J). Co-IP experiments revealed that LPS treatment significantly reduced the interaction between HKDC1s and HSCBs compared with that in the control group. This process that resulted in the binding of HSCB to FDX1 was significantly reduced under LPS stimulation ( Figure 3K). These protein-protein interactions suggest that the interactions among HKDC1, FDX1, and HSCB may influence the expression or function of FDX1, thereby altering cellular copper ion homeostasis and ultimately regulating cuproptosis. In summary, LPS blocks the cuproptosis pathway by activating HKDC1-mediated glycolysis and disrupting the HKDC1-related protein interaction network.

### LPS mediates the secretion of inflammatory cytokines via HKDC1

**Figure 4:**
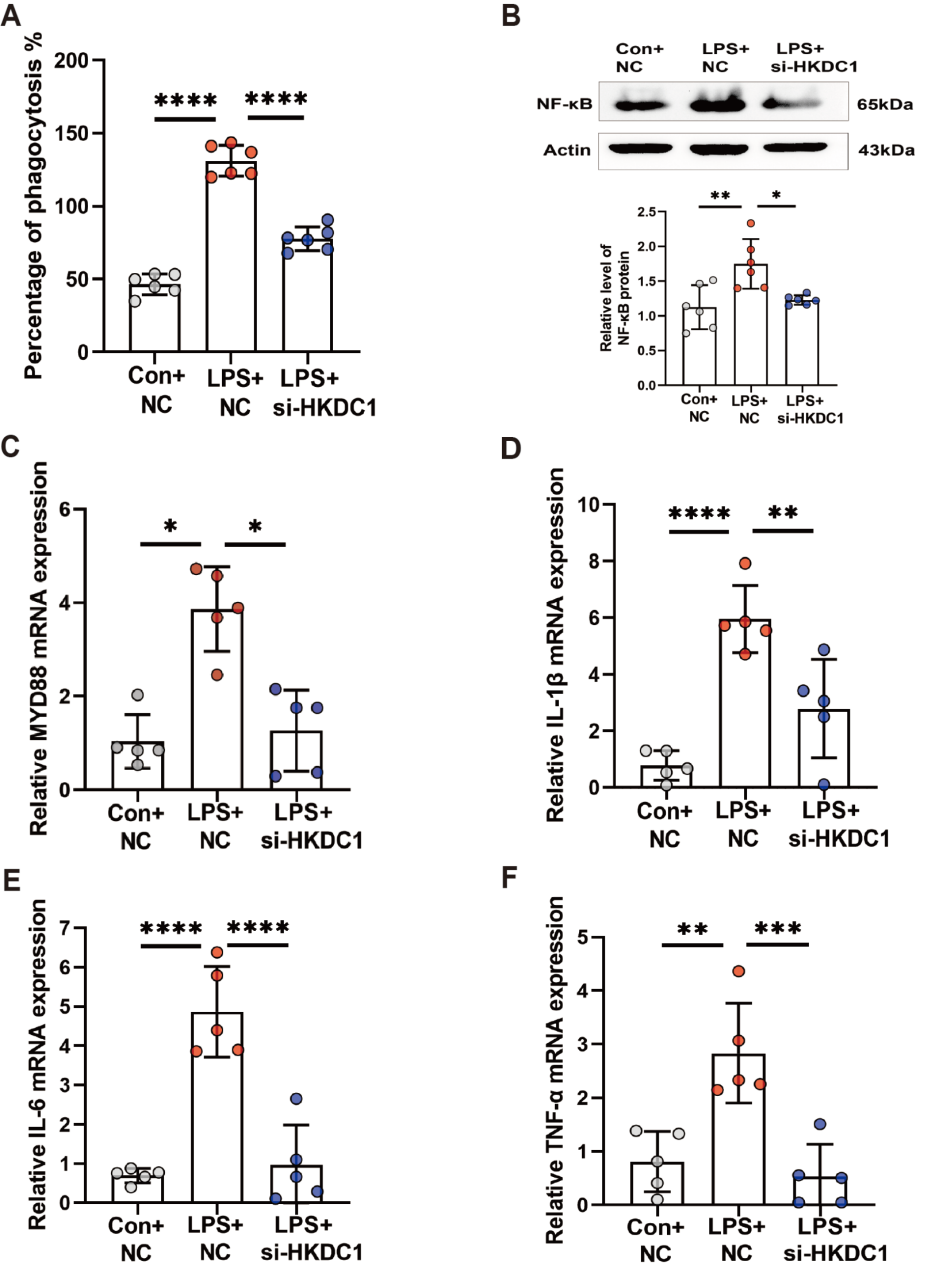
Figure 4 si-HKDC1 reverses the LPS-induced inflammatory response (A) Phagocytosis assay was used to detect the phagocytic activity of si-HKDC1-treated cells (n = 6). (B) Western blot analysis was used to detect the protein expression of NF-κB after si-HKDC1 treatment (n = 6). (C–F) RT-qPCR was used to detect the mRNA expressions of MYD88, IL-1β, IL-6, and TNF-α after si-HKDC1 treatment (n = 5). Data are presented as the mean ± SD. One-way ANOVA with Tukey’s post hoc test was used for multiple comparisons. Con, control. NC, negative control. si-HKDC1, siRNA-mediated HKDC1 knockdown. *P < 0.05, **P < 0.01, *** P < 0.001, ****P < 0.0001.

To further clarify whether HKDC1 can mediate the inflammatory response through cuproptosis, we first detected the phagocytic function of THP-1 cells. The data revealed that the phagocytic function of the cells treated with si-HKDC1 was decreased compared with that of the LPS-treated cells ( Figure 4A). These results confirm that the LPS/TLR4 pathway can regulate inflammation-related pathways and inflammatory responses via HKDC1. Western blot analysis experiments demonstrated that transfecting THP-1 cells with si-HKDC1 reduced the expression of the NF-κB protein induced by LPS ( Figure 4B). We subsequently detected the expressions of genes associated with the inflammatory pathway. RT-qPCR experiments revealed that treatment with LPS in combination with si-HKDC1 significantly reduced the mRNA levels of *MYD88*, *IL-1β* , *IL-6*, and *TNF-α* in the cells ( Figure 4C‒F). These results confirm that LPS can regulate inflammation-related pathways and inflammatory responses through HKDC1.

### LPS regulates HKDC1 expression through YY1

**Figure 5:**
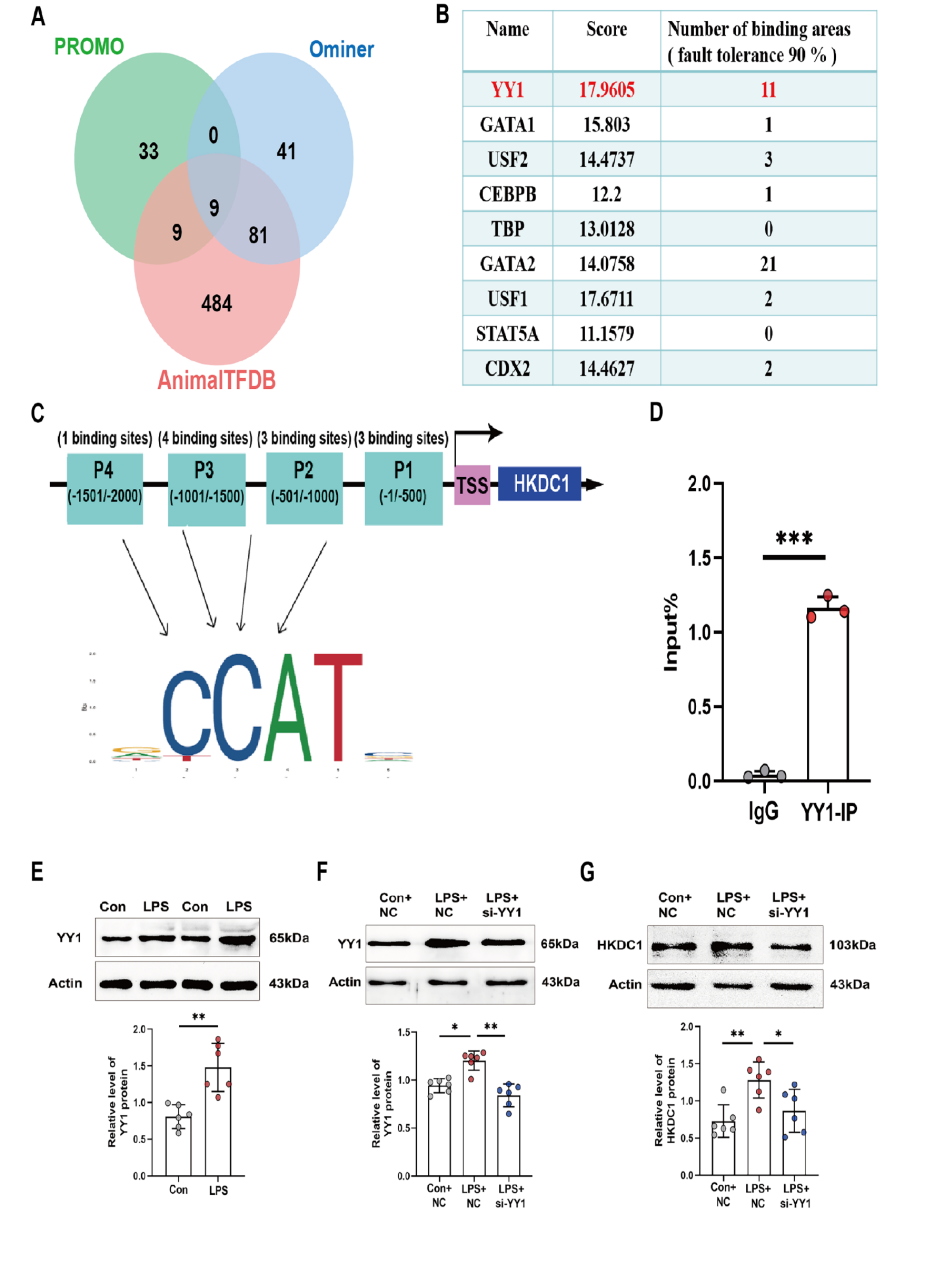
Figure 5 LPS induces YY1 to regulate HKDC1 expression (A,B) The PROMO ( https://alggen.lsi.upc.es/cgibin/promo_v3/promo/promoinit.cgi?dirDB=TF_8.3), Ominer ( http://signalingpathways.org/ominer/query.jsf) and AnimalTFDB (https://ngdc.cncb.ac.cn/databasecommons/database/id/8) databases were used to screen for transcription factors that bind to the HKDC1 promoter. (C) The binding sites and motifs of YY1 within HKDC1 promoter fragments were predicted by JASPAR ( https://jaspar2022.genereg.net/docs/). (D) ChIP-qPCR was used to detect the binding of the HKDC1 promoter with YY1 (n = 3). (E,F) Western blot analysis was performed to assess YY1 protein expression and the interference efficiency of si-YY1 (n = 6). (G) The expression of HKDC1 after si-YY1 treatment was also evaluated (n = 6). Data are presented as the mean ± SD. One-way ANOVA with Tukey’s post hoc test was used for multiple comparisons, and independent samples t tests were employed for pairwise comparisons. Con, control. NC, negative control. si-YY1, siRNA-mediated YY1 knockdown. Input, whole-cell lysate. IgG, non-specific binding control. IP, DNA-protein complex pulled down by antibodies against YY1. TSS, Transcription start site. *P < 0.05, **P < 0.01, ***P < 0.001.

To elucidate the potential upstream mechanism of LPS-induced HKDC1 expression, we predicted 9 transcription factors that may bind to the *HKDC1* promoter using the PROMO, Ominerh, and AnimalTFD databases. Among these genes, *YY1* (Yin Yang 1) had the highest binding score ( Figure 5A,B). Moreover, the *HKDC1* promoter region was equally divided into four DNA fragments. The binding sites and representative motifs of YY1 within the *HKDC1* promoter were predicted using the JASPAR database ( Figure 5C). The ChIP-qPCR results verified that YY1 was recruited to the *HKDC1* promoter fragment ( Figure 5D). The western blot analysis results revealed that LPS promoted the expression of YY1 ( Figure 5E). Next, we transfected THP-1 cells with either NC or si-YY1 and confirmed that si-YY1 significantly inhibited LPS-induced YY1 expression ( Figure 5F). Finally, we verified that si-YY1 transfection significantly reduced HKDC1 expression levels in macrophages following LPS treatment ( Figure 5G). These results confirm that LPS induces YY1 to bind to the *HKDC1* promoter and regulate its expression.

### HKDC1 regulates LPS-induced sepsis in mice

**Figure 6:**
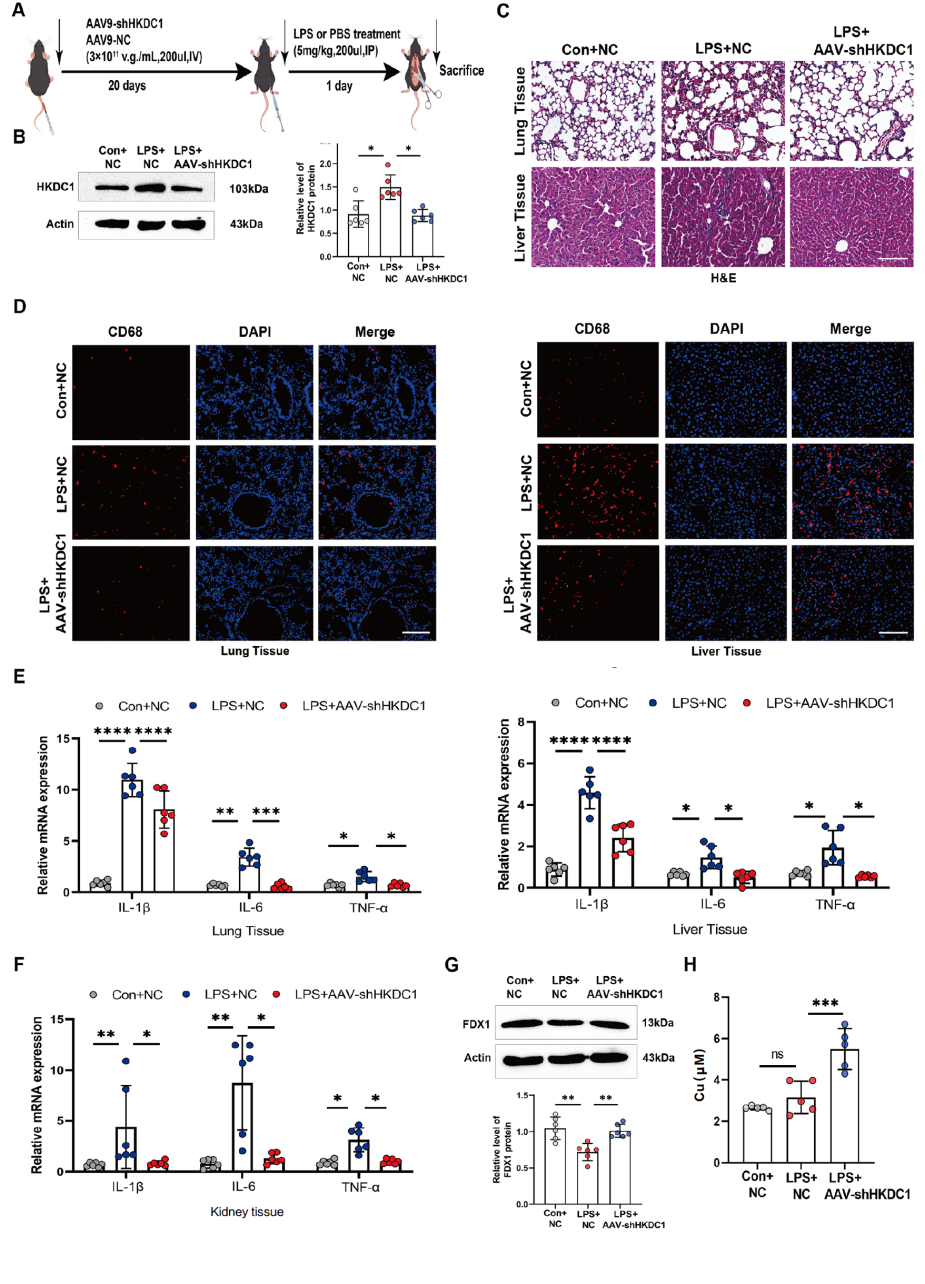
Figure 6 Knockdown of *HKDC1* can alleviate sepsis in mice (A) Mice were intravenously injected with AAV9 once. After 20 days of standard feeding, the mice were intraperitoneally injected with LPS for 1 day (n = 10). (B) Western blot analysis was used to detect the protein expression of HKDC1 in mouse lung tissue (n = 6). (C) Pathological changes in the lungs and livers of the mice were evaluated using H&E staining ( n = 3); scale bar, 100 μm. (D) Tissue fluorescence assays were used to detect CD68-positive macrophages in mouse lung and liver tissues ( n = 3); scale bar, 100 μm. (E,F) RT-qPCR was used to detect the mRNA expressions of IL-1β, IL-6, and TNF-α in the lung, liver, and kidney tissues of the mice (n = 6). (G) Western blot analysis was used to detect the protein expression of FDX1 in the lung tissue of the mice (n = 6). (H) Copper ion levels were detected in the lung tissue of the mice (n = 5). Data are presented as the mean ± SD. One-way ANOVA with Tukey’s post hoc test was used for multiple comparisons, and independent samples t tests were employed for pairwise comparisons. Con, control. NC, negative control. AAV-shHKDC1, adeno-associated virus short hairpin RNA targeting HKDC1. ns not significant, *P < 0.05, **P < 0.01, ***P < 0.001, ****P < 0.0001.

To evaluate the role of HKDC1 in inflammatory diseases *in vivo*, we established an LPS-induced mouse sepsis model on the basis of protocols described in the literature [ 31, 32 , 41, 42] ( Figure 6A). Western blot analysis revealed that the protein level of HKDC1 was significantly increased in the lung tissues of LPS-treated mice, whereas AAV-shHKDC1 treatment mitigated this effect ( Figure 6B). H&E staining revealed that, compared with the control, treatment with LPS resulted in significant damage to the lung and liver, as characterized by cell swelling and inflammatory infiltration ( Figure 6C). The fluorescence staining results revealed a significant reduction in the number of CD68-positive macrophages in the liver and lung tissues of the AAV-shHKDC1 group ( Figure 6D). RT-qPCR analysis revealed that AAV-shHKDC1 treatment significantly decreased the mRNA levels of the proinflammatory cytokine genes *IL-1β*, *IL-6*, and *TNF-α* in mice induced with LPS ( Figure 6E,F). Finally, we detected cuproptosis-related indicators and found that the protein level of FDX1 increased after AAV-shHKDC1 treatment ( Figure 6G). Copper content measurements revealed a slight, non-significant increase in copper levels in the liver tissues of the LPS-treated mice ( *P* > 0.05). In contrast, the copper content of the AAV-shHKDC1 group significantly increased, suggesting that *HKDC1* knockdown may promote intracellular copper accumulation ( Figure 6H). These results suggest that HKDC1 plays a significant role in the LPS-induced inflammatory response by regulating cuproptosis.

## Discussion

**Figure 7:**
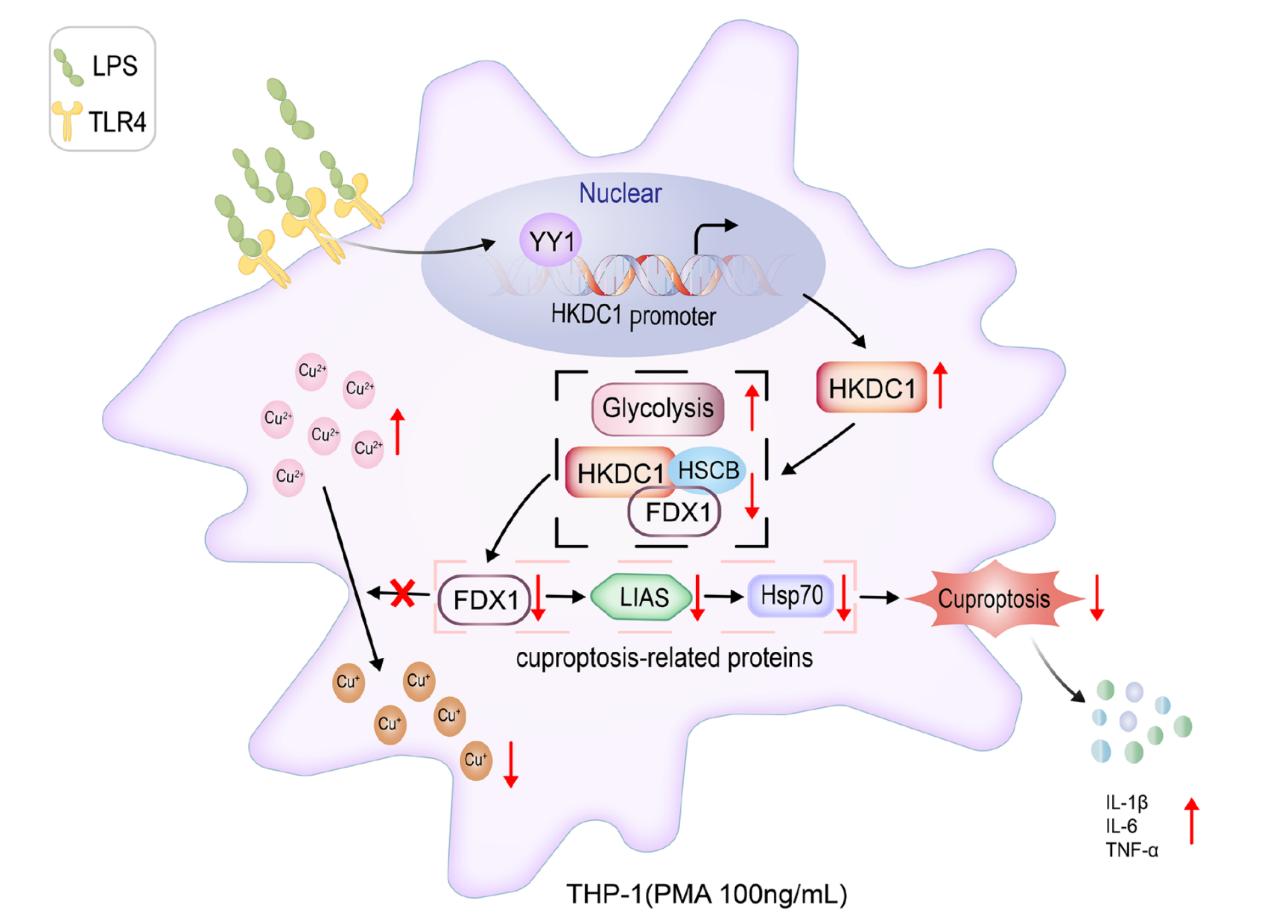
Figure 7 LPS promotes the inflammatory response in THP-1 macrophages by inhibiting cuproptosis via HKDC1 Initially, LPS binds to the TLR4 receptor on the cell membrane. In the nucleus, YY1 is activated and subsequently binds to the HKDC1 promoter region, promoting its transcription and translation. The produced HKDC1 protein activates the glycolytic pathway in the cytoplasm and concurrently reduces complex formation between HKDC1, HSCB, and FDX1, leading to decreased expression or function of FDX1. This cascade ultimately inhibits cuproptosis and promotes the release of inflammatory factors in THP-1 macrophages.

In this study, we revealed a novel regulatory mechanism of cuproptosis by utilizing LPS-induced THP-1 macrophages and animal models of inflammation. The experimental results demonstrated that HKDC1 mediates LPS-induced cuproptosis and exacerbates the inflammatory response, as illustrated in Figure 7. LPS is recognized by the TLR4 receptor on the cell membrane of THP-1 cells. Within the nucleus, the transcription factor YY1 binds to the *HKDC1* promoter, thereby promoting its transcription. In the cytoplasm, HKDC1 activates the glycolytic pathway. Concurrently, the interaction among HKDC1, HSCB, and FDX1 results in reduced expression or function of the cuproptosis-related gene FDX1 and its downstream proteins LIAS and Hsp70. This interaction prevents the reduction of Cu ^2+^ to Cu ^+^, thereby protecting cell viability and phagocytic function. Ultimately, this process stimulates the release of inflammatory factors.

Macrophages perform functions such as engulfing foreign pathogens and endogenous cell debris, presenting signal molecules, and activating the immune response, which play key roles in tissue development and homeostasis [43]. The gene expression profile of LPS-stimulated THP-1 cells closely resembles that of primary peripheral blood mononuclear cell (PBMC)-derived macrophages, making them a suitable model for sepsis and inflammation studies [44]. Previous studies have demonstrated that the co-activation of macrophages with LPS and IFN-γ stabilizes the HIF-1α subunit and induces metabolic reprogramming toward glycolysis in M1 macrophages [ 45 , 46]. Similarly, in this study, THP-1 macrophages were induced with LPS at a concentration of 100 ng/mL for 24 h, leading to a significant increase in glycolysis and the release of proinflammatory cytokines, including IL-1β, IL-6, and TNF-α. However, macrophages treated with different concentrations of LPS for different durations may exhibit varying degrees of functional alterations [47]. In our study, we determined that 100 ng/mL was the optimal LPS concentration, as it corresponded to the highest cell viability for validation. Therefore, further exploration is needed to assess the effects of different LPS concentrations and various treatment durations on THP-1 macrophage glycolysis and the inflammatory response.

Cuproptosis is a recently discovered form of regulated cell death triggered by excess Cu ^2+^ [ 19, 20, 48]. Previous studies have reported that tumor cells that utilize glycolysis as the primary metabolic pathway for energy production are tolerant to cuproptosis, whereas those that rely on the tricarboxylic acid cycle and oxidative phosphorylation are more sensitive [19]. Although elevated levels of Cu ^2+^ are found in multiple tumor tissues and serum, key cuproptosis genes, including *FDX1*, *SDHB*, *DLAT*, and *DLST*, are downregulated in tumor tissues [20]. This leads to blockade of the cuproptosis pathway, resulting in increased migration and proliferation of tumor cells [20]. In a proinflammatory environment, combining Cu ^2+^ and low concentrations of anti-inflammatory agents promotes macrophage inflammation [ 49, 50]. In this study, we found that LPS stimulation inhibited cuproptosis but did not significantly promote the production of Cu ^2+^ at the cellular or animal level ( Figure 1D and Figure 6 H; *P* > 0.05). This result is consistent with those of previous studies, which revealed that high concentrations of LPS and Cu ^2+^ can induce M1 gene expression in THP-1 macrophages, whereas moderate to low concentrations of Cu ^2+^ increase TNF-α expression [ 51, 52]. Moreover, our observations were consistent with those of Tsvetkov *et al*. [20], who reported that cytotoxicity and cell death are triggered only when the concentration of Cu ^2+^ is extremely high. We found that LPS alone failed to increase the concentration of Cu ^2+^ to toxic levels. In contrast, LPS combined with *HKDC1* knockdown significantly increased Cu ^2+^ levels, promoted cuproptosis, and inhibited the release of inflammatory factors. This effect may be attributed to *HKDC1* knockdown, which blocks LPS-induced glycolysis and certain inflammatory signaling pathways [ 53 , 54], thereby promoting a shift in cell metabolism toward the tricarboxylic acid (TCA) cycle [ 55– 57]. Additionally, combined treatment with LPS and *HKDC1* knockdown increased the expression or function of the cuproptosis-related gene *FDX1*. This, in turn, leads to an increase in toxic Cu ^+^ levels, inducing cell death and inhibiting the inflammatory response. More importantly, the changes in copper transporters, specifically ATPase copper transporting beta (ATP7B) and solute carrier family 31 member 1 (SLC31A1), in LPS-stimulated THP-1 macrophages remain unclear and require further investigation [ 17, 49] .

In response to LPS stimulation, macrophage metabolism is driven predominantly by the anaerobic glycolytic pathway [58]. Consistent with these findings, the results of this study revealed that the expressions of HK2 and PKM2, as well as the PA content, were increased, whereas the activity of PDH was decreased in THP-1 macrophages under LPS induction. To further elucidate the mechanism of cuproptosis regulation, we focused on *HKDC1*, a key glycolytic gene that is overexpressed in certain cancers and is the fifth hexokinase discovered in recent years [59]. HKDC1 promotes tumorigenesis and glycolysis in lung adenocarcinoma (LUAD) by regulating the AMPK-mTOR pathway, and targeting HKDC1 can delay LUAD progression [ 15, 16]. Additionally, HKDC1 facilitates tumor immune escape by recruiting cytoplasmic STAT1 to IFNGR1, thereby revealing its critical role in the tumor immune microenvironment and providing theoretical support for combined immunotherapy [54]. This study demonstrates for the first time that the LPS-induced transcription factor YY1 binds to the *HKDC1* promoter, increasing its expression in THP-1 macrophages. After *HKDC1* was knocked out, the expressions of the LPS-inhibited cuproptosis-related proteins FDX1, LIAS, and Hsp70 were increased, whereas the expressions of the inflammatory pathway components MYD88, IL-1β, IL-6, and TNF-α were decreased. These findings reveal a novel function of HKDC1 in glycolysis, copper homeostasis, and inflammation regulation. Although we identified HKDC1 as a potential anti-inflammatory target, HKDC1 inhibition in our studies was achieved through gene interference or adenovirus transduction. Because there are currently no commercially available inhibitors or agonists of HKDC1, large-scale preclinical studies cannot be conducted. Future research should prioritize the development of small-molecule inhibitors and agonists that can specifically bind to the active sites or key domains of HKDC1, thereby modulating its function and providing more possibilities for clinical research. On the basis of our findings, targeting HKDC1 may represent a novel strategy for treating inflammation by selectively inhibiting the metabolism and proliferation of inflammatory cells. However, more evidence is needed to clarify whether HKDC1 functions beyond its role as a hexokinase in regulating cuproptosis.

This study is limited in that it did not investigate the expression and function of HKDC1 in other inflammatory models, particularly in various immune cells, such as primary macrophages, neutrophils, NK cells, and T cells. The metabolic activity of immune cells, especially glycolysis, is intricately linked to the intensity and outcome of the immune response [ 60, 61]. For example, LPS induces an inflammatory response in RAW264.7 cells via the mitogen-activated protein kinase (MAPK) signaling pathway [ 62, 63]. The tumor necrosis factor-alpha/tumor necrosis factor receptor 2 (TNF-α/TNFR2) axis promotes aerobic glycolysis in NK cells, thereby enhancing the immune response [ 64, 65]. LPS enhances neutrophil glycolysis via the phosphoinositide 3-kinase/protein kinase B/hypoxia-inducible factor 1 alpha (PI3K/Akt/HIF-1α) pathway, thereby providing sufficient energy to support phagocytosis and chemotaxis [66]. These results suggest that glycolysis and its associated genes play crucial roles in energy metabolism during immune responses. Therefore, further investigation into the expression and function of HKDC1 in a broader range of immune cells is essential for elucidating its potential role in the inflammatory response.

In summary, our findings demonstrated that LPS exacerbated glycolysis and cellular cuproptosis resistance by promoting HKDC1 expression, which effectively increased cell viability and ultimately led to a proinflammatory response in THP-1 macrophages. Thus, for the first time, we confirmed the expression of HKDC1 in LPS-induced THP-1 cells and acute sepsis mouse models, elucidated its role in cuproptosis and inflammation at both the cellular and animal levels, and established a potential target for the treatment and diagnosis of inflammatory diseases.
